# Enolase 1, a Moonlighting Protein, as a Potential Target for Cancer Treatment

**DOI:** 10.7150/ijbs.63556

**Published:** 2021-09-21

**Authors:** Gan Qiao, Anguo Wu, Xiaoliang Chen, Ye Tian, Xiukun Lin

**Affiliations:** 1 School of Pharmacy, Southwest Medical University, Luzhou, 646000, China (Q.G, dqz377977905@swmu.edu.cn).; 2 School of Pharmacy, Central Nervous System Drug Key Laboratory of Sichuan Province, Southwest Medical University, Luzhou, 646000, China.; 3 Sichuan Key Medical Laboratory of New Drug Discovery and Drugability Evaluation, Luzhou Key Laboratory of Activity Screening and Drugability Evaluation for Chinese Materia Medica, School of Pharmacy, Southwest Medical University, Luzhou, 646000, China.; 4 Education Ministry Key Laboratory of Medical Electrophysiology, Southwest Medical University, Luzhou, 646000, China.; 5 Schools of Medicine; Shanxi Datong University, Datong, Shanxi, 037009, China.; 6 The Eighth Affiliated Hospital Sun Yat-sen University,Shenzhen, Guangdong, China.; 7 College of Life Sci., Shandong University of Technology, Zibo, Shandong, China.

**Keywords:** Enolase 1, Moonlighting Protein, transduction cascades, Cancer treatment

## Abstract

Enolase 1 (ENO1) is a moonlighting protein, function as a glycolysis enzyme, a plasminogen receptor and a DNA binding protein. ENO1 play an important role in the process of cancer development. The transcription, translation, post-translational modifying activities and the immunoregulatory role of ENO1 at the cancer development is receiving increasing attention. Some function model studies have shown that ENO1 is a potential target for cancer treatment. In this review, we provide a comprehensive overview of the characterization, function, related transduction cascades of ENO1 and its roles in the pathophysiology of cancers, which is a consequence of ENO1 signaling dysregulation. And the development of novels anticancer agents that targets ENO1 may provide a more attractive option for the treatment of cancers. The data of sarcoma and functional cancer models indicates that ENO1 may become a new potential target for anticancer therapy.

## Introduction

As a moonlight protein, Enolase 1 (ENO1) performs multiple biochemical functions. ENO1 catalyzing 2-phosphoglycerate (2-PGA) to phosphoenolpyruvic acid (PEP), a vital step in the glycolysis, plays an important role in several pathophysiological processes 1. ENO1, anchored on the cell membrane, servers as a receptor for activating plasminogen to stimulates the migration and invasion ability of cells 2. Furthermore, ENO1 also displays multiple binding capacity to the DNA, mRNA 3, LncRNA and tRNA(CUU)Lys to regulate the gene transcription and translation in cancer cells 4,5. Since the energy of cancer cells mainly rely on the glycolysis, so that ENO1 has been developed as an important target in cancer treatment.

The expression levels of ENO1 are closely associated with several diseases, including Alzheimer's disease 6, diabetes 7, hypoxic-ischemic encephalopathy 8, as well as some types of cancers 9-16. ENO1 has been found to be overexpressed in more than 10 types of human cancer, and is related with metabolic reprogramming of cancer cells and cancer associated transduction cascades. And, the expression of ENO1 in a variety of cancer types have been found to be dependent on the pathophysiological conditions. In the cancer metabolic reprogramming, ENO1 stimulates cancer cells to create energy largely by disintegration of glucose in a non-oxidative manner rather than typical oxidative phosphorylation 17,18. The switching of ENO1 location is related to the pathology of cancer and inflammatory. The inflammatory stimulation could induce ENO1 translocation from the cytosolic to the cell membrane 7,19.

As the important pathophysiological biomarker in the cancers, targeting ENO1 or ENO1-mediated signaling pathways has been received the most attention. Over the years, several ENO1 inhibitors have performed promising results of the pharmacological-based study and preclinical trials 20,21. As the non-enzymatic active site inhibitor of ENO1, ENOblock reflects cytotoxicity in hypoxic and normal conditions on colon cancer cells^22^. In addition, a series of enzymatic active inhibitors of ENO1, such as phosphonoacetohydroxamate acid (PhAH), (1,5-dihydroxy-2-oxopyrrolidin-3-yl)phosphonic acid (SF2312), deoxy-SF2312, Methyl-SF2312, POMSF, POMHEX and its derivatives, show potential anticancer in the functional cancer models 21,23. Furthermore, overexpression of ENO1 contributes to immune response and autoimmunity in the patients 24-27. The recently studies show that ENO1 is also an effective target for anticancer immunotherapy 28. ENO1 binds with guanylate binding protein to negative regulate T cell signaling by interfering with early T Cell Receptor signaling 29. And the post-translational modifying activities is also critical to the function of ENO1 in the immunity 26,30,31. A landmark study suggests that citrullinated ENO1 peptides could be an effective vaccines for cancer immunotherapy 32. In our previous study, we found that granulin A inhibit the cell invasion and migrating by interacting with ENO1. However, the catalytic activity of ENO1 and the glucose uptake in the hepatocellular carcinoma (HCC) cells is enhanced by granulin A. And, The agents targeting ENO1 are attracting attention as a novel cancer therapeutics 33.

With the novel findings in several other publications, a careful review and reanalysis of published finding on ENO1 is necessary. Herein, we mainly review the characterization, function, transduction cascades and inhibitors of ENO1 for depicting a detail portrait of ENO1 in the cancer development.

## The structure and function of ENO1

The cytogenetic location of *ENO1* is located at the locus 1p36.23. The *ENO1* gene spans about 18 kb and consists of twelve exons and eleven introns. Hypoxia-response elements (HREs), the sequence NCGTG, are located in the ENO1 promoter. The expression of ENO1 is also regulated by hypoxia-inducible factor 1 (HIF-1) recognizing to HREs in the genome for responding the hypoxia stress 34. Because of *ENO1* gene lacks the canonical TATA box, so that the *ENO1* possessing multiple transcription starts sites 35. The *ENO1* gene encodes both ENO1 and c-myc binding protein (MBP-1), and mounting shreds of evidence have indicated that both ENO1 and MBP-1 play pivotal roles in tumorigenesis. The isoform of ENO1 gene, MBP-1 expression is regulated by the protein kinase B (AKT)/ eukaryotic initiation factor 2α (eIF2α) transduction cascades to response cell stress. The study implies that alternative splicing of ENO1 mRNA could be controlled by the AKT/eIF2α axis 36. There are three flexible active site loops of ENO1 are named as L1 loops (residues 36-43), L2 loops (residues 156-162) and L3 loops (residues 262-270) in crystal structures of ENO1, respectively^37^. And two magnesium ions bind to Glu292, Asp317, Asp244 and Ser39, they are the key active site residues of ENO1. In these studies, the target drug is always interacting with the activity residues of ENO1.

As moonlighting protein, ENO1 performs distinct functions and involves in multiple cellular processes in cells. As phosphopyruvate hydratase, ENO1 participates in the 9^th^ step of glycolysis and is responsible for the catalysis of the conversion of 2-PGA to PEP, which in turn is converted by pyruvate kinase to pyruvate. The Ser41 of ENO1 show closer to the phosphate group of the 2-PGA and directly interacts with the second Mg^2+^ of ENO1 38. As a plasminogen receptor, ENO1 converts plasminogen to plasmin, which involves in the metastatic spread of cancer from the primary tumor to a remote site. In the intravascular and pericellular fibrinolytic system, ENO1 activates plasminogen (PLG) on the cell surface of several cell-types, such as leukocytes and neurons 39. The Lys345, Glu211 and the metal cations of ENO1 forms an essential part of the reaction mechanism to activate the plasminogen. The Lys345 of ENO1 plays an important role in capturing the R-proton of plasminogen, and then Glu211 is protonated and hydrogen bonded to the a-hydroxyl group of plasminogen 40. The putative plasminogen-binding motif of ENO1 (residues: 250-256) and three hydrophobic and two hydrophilic residues play a critical role in the enzyme capacity of ENO1 1. As a DNA binding protein, the residues of 97-237 are required for binding capacity of ENO1 with c-Myc promoter to repress tumorigenic 41.

## Post-translational modifications of ENO1 play an important role in the function change of ENO1

Post-translational modifications (PTMs) of ENO1 are the important mechanism of regulation of protein function and cellular transduction cascades events that orchestrate biological functions in mammalian cells. Acetylation, phosphorylation, succinylation, hydroxyisobutyrylation (Khib) and methylation of ENO1 can alter the catalytic activity, localization, protein stability and bounding capacity of ENO1. Investigation of these modification patterns in different human cancer cells have provided insights into its important role in pathophysiological processes 42. As shown in the **Table 1**, the 44 modified residues of ENO1 have been mention, identified and investigated in the decades.

The enzyme activity of ENO1 is always modulated by the various PTMs in the glycolysis. Acetylation modified of protein is one of the major PTMs 43,44. Acetylation lysine residue 335 of ENO1 by histone deacetylase 11 (HDAC11) causes loss of ENO1 activity and suppress the glycolysis in tumor cells 64. Acetylation lysine residues 257 and 283 of ENO1 could disrupt both the electrostatic binding potential and the geometry of the binding site and perturbing substrate binding capacity of ENO1 to inhibited the glycolysis in *Synechococcus elongates* 45,65. Moreover, the enzyme activity of ENO1 and others are regulated by acetylation and succinylation modified in metabolism, thus mediating the anticancer drug effect of dichloroacetate is related with the glycosis 66. The succinylation and desuccinylation of ENO1 is co-regulated by carnitine palmitoyltransferase I (CPT1A) and sirtuin 5 (SIRT5), respectively. Kiran Kurmi et al. indicate that CPT1A inhibits enzymatic activity of ENO1 by succinylation of ENO1 to promote hematopoietic cell proliferation under glutamine depletion. And the succinylation of ENO1 lysine residues 80, 81 and 335 are also the important sites for the catalyst activity of ENO1 49,67. Khib may directly mediate environmental influences on the epigenome and biological processes. To regulate the glycolysis of colorectal carcinoma cell HCT116, the surface lysine residues of ENO1 could be Khib modified by p300 protein 60. However, the NAD-dependent protein deacylase (CobB) regulates the catalytic activities of ENO1 by removing ENO1K343hib and ENO1K326ac simultaneously in prokaryotes. That meaning that the Khib of ENO1 play an important role of regulating of cellular glycolysis and cell growth 48. Previous studies have shown that phosphoglycerate kinase (pgK) of metabolism enzymes play an important role of regulating the glycolytic process and glucose metabolism 68-70. Enhancing pgK modification of ENO1K343 causes the decreasing of enzyme activities of ENO1 61. The acetylation, succinylation, Khib and pgK modification play critical role for the catalysis capacity of ENO1 in the glycolysis.

As a direct substrate of the serine/threonine-protein kinase (ULK1), the phosphorylation of ENO1 protect cells from ROS-associated cell death 54. Furthermore, ENO1 phosphorylation is also regulated by JAK-independent signaling controlled PIP3 levels and AKT activity in cytotoxic T cells 56. Arginine residues 50 of ENO1 is methylated by protein arginine methyltransferase 5 (PRMT5) that plays an important role for increasing cell invasion and migration in cancer 30. The interferon (IFN)-stimulated gene product 15 modification (ISGylated) of ENO1 is found in the human lung adenocarcinoma epithelial cell line A549 and other bacterial 46,62,63, however the function of ISGylated ENO1 remain unclear. The PTMs of protein is closely related with the function and structure of protein, the details of PTMs of ENO1 might reveal some function characterization in the change of structure. As shown in the **Table 1**, the single residue might be different modified types. And the most modifications of ENO1 are related with the substrate binding capacity, catalyst activity, cellular glycolysis and cell invasion and migration in cancer.

## ENO1 participates in the important transduction cascades of cancer

### Hypoxia-induced ENO1 expression

Hypoxia microenvironment leads to the expansion of aggressive clones of heterogeneous tumor cells, and promotes a lethal phenotype in the tumor 71. ENO1 is required for maintaining the Warburg effect of cancer cells. ENO1 responds to the hypoxic microenvironments in the rapid proliferation cells. HREs of *ENO1* gene is a key transcription element in the expression of ENO1. The H3K9 acetylation at HREs of *ENO1* gene are significantly increased by mucin-1 (MUC1) overexpression in a hypoxia-dependent manner, and the mRNA of ENO1 show a significant increase 72. Moreover, ENO1 also is temporally distinct transcriptional regulation by HIF1α-HIF1β binding with HREs of *ENO1* to increase the expression of ENO1 73,74. Furthermore, the expression of ENO1 and other glycolysis genes also can be regulated by sineoculis homeobox homolog 1 (SIX1), which interact with acetyltransferase 1 (HBO1) and Nuclear receptor coactivator 3 (AIB1) to affect Warburg effect in cancer 75. In addition, SIX1 glycolytic function is directly repressed by microRNA-548a-3p. The miR-548a-3p/SIX1 axis regulates aerobic glycolysis by alteration expression of glycolysis genes in cancer cells 75. Knockdown of ENO1 increases ROS as well as tricarboxylic acid (TCA) cycle activity, induces a decline in nucleotide base synthesis and promotes cellular senescence mainly through the sorbitol in cancer cell lines 76. Zhang et al. indicate that the phosphoglycerate mutase-enolase-pyruvate kinase interacts with voltage-dependent anion channel protein (VDAC1) of the outer mitochondrial membrane and the triose phosphate translocator of the chloroplast, and the interaction is considerably more efficient than the free enzymes of the extra-plastidial preparation, as well as prevents the use of substrate by competing reactions such as phosphoenolpyruvate carboxylase and amino acid biosynthesis in the plant. And lacking enolase compromised the movement of their mitochondria as well as the degree of mitochondria-chloroplast colocalization, so that the interaction of enolase with mutase/pyruvate kinase are important for promoting a highly efficient coordination of the major energy systems of the plant cell 77. However, mitochondrial ENO1 interacting with VDAC1 prevents Ca^2+^-induced loss of mitochondrial transmembrane potential, swelling of matrix and release of cytochrome c to regulate cardiomyocyte apoptosis, a critical regulator of the mitochondrial cell death pathway in the mammalian cells 78. The interaction of ENO1 with others glycolysis enzymes should be clarified in the mammalian, that may be a critical mechanism for the tumorigenesis.

Elevation of ENO1 could promotes glycolysis, cellular proliferation and migration via activating the MAPK/ERK pathways. Dr. Chen et al. show that cytotoxin-associated gene A (CagA) activates the Src and MAPK/ERK to induce the elevation of expression of ENO1 in *H. pylori*-mediated gastric cancer. U0126 (selective inhibitor of both MEK1/2) and PP1 (inhibitor of Src kinase) can attenuate the upregulation of ENO1 in the wild-type H. pylori CagA^+^-infected cell 79.

### ENO1 and PI3K/AKT

AKT is a serine/threonine kinase that is involved in mediating various biological responses, such as inhibition of apoptosis and proliferation. The phosphoinositide 3-kinase (PI3K)/AKT signaling pathway is the major pathway for driving cancer cells to favor glycolysis over mitochondrial oxidation. Sun *et al*. demonstrate that ENO1 is a positive regulator of the PI3K/AKT axis for promoting gastric cancer cell proliferation and metastasis 80. Otherwise, Chen et al. elucidate that ENO1/AKT/PI3K signaling axis is mediates by WW Domain Binding Protein 2 (WBP2) modulating the expression and glycolysis activity of ENO1 to regulate the proliferation and metastatic ability of glioma cells 81. Furthermore, overexpression of ENO1 significantly increase the levels of β-catenin and phosphorylated focal adhesion kinase (FAK), PI3K, and AKT. They indicate that ENO1 is an upstream signal factor modulating the FAK/PI3K/AKT axis in non-small-cell lung carcinoma (NSCLC) to promote glycolysis, proliferation, migration and invasion of cancer cells 82. ENO1 contribute to the subsequent cellular response of cancer and may be a critical effector in PI3K/AKT pathway.

### ENO1 and AMPK/mTOR

AMP-activated protein kinase (AMPK) respond on the external stress by gauging the AMP and ATP levels of the cell. In cancer, ENO1 induces the ATP production in HCT116 cells. Furthermore, ENO1 also promotes growth, tumorigenesis, migration, invasion and metastasis of colorectal cancer cells by AMPK/mTOR pathway. Mechanically, ENO1 inhibits phosphorylation of AMPKα while increases the phosphorylation of mTOR concomitantly to promote tumorigenesis and metastasis 83. However, the overexpression of ENO1 regulate proliferation, de-differentiation, resistance to apoptosis and the hypoxia-induced metabolic shift via the AMPKα1/AKT/GSK3β in human pulmonary artery smooth muscle cells (PASMC). ENO1 up-regulate phosphorylation levels of AKT (T308 and S473) and GSK3β via the major kinase AMPK 84. Phosphorylated AMPK (T172) is observed in ENO1-deleted D423 glioma cell line, which induce growth inhibition and subsequent apoptosis 23,85.

### ENO1 and Wnt/β-catenin

Canonical Wnt signaling controls key developmental gene expression programs by increasing nuclear and cytoplasmic β-catenin. Some studies demonstrate that ENO1 functions as an oncogene in bladder cancer to regulate cell cycle and apoptosis by regulating β-catenin. Overexpression of ENO1 can up-regulate β-catenin as well as its downstream targets cyclin D1 to promotes cell growth and proliferation 82,86. In addition, ENO1, protein disulfide isomerase family A member 3 (PDIA3) and podoplanin (T1α) involved in β-catenin driven trans-differentiation of murine alveolar epithelial cells. The Wnt10a and Wnt10b represent potential Wnt ligands to active Wnt/β-catenin signaling. Activated Wnt/β-catenin signaling regulates expression of ENO1, PDIA3 and carbonyl reductase 2 (CBR2) to induce the ATII-to-ATI cell trans-differentiation 87.

### ENO1 and LncRNA

The expression regulation and dysfunction of ENO1 have a closely relationship with the hypoxic metabolic environment in the cancer development. And the long non-coding RNA is also an important regulator for the expression of ENO1. Dr. Liu, et al., find that LncRNA P5848 is upregulated in the mimic environment of radiofrequency ablation. LncRNA P5848 promotes HCC development by upregulating ENO1. Comparatively, blocking of LncRNA P5848/ENO1 axis can attenuate growth, survival and invasion capacity of cancer cells 12. However, the other LncRNA-6195 reduces enzymatic activity of ENO1 to represses the growth of HCC via directly binding with ENO1 13.

### ENO binding proteins and the MBP-1 related pathway

ENO1 is able to activate the CD14-dependent TLR4 pathway via binding with TLR4 on monocytes involving in a dual mechanism firstly pro-inflammatory and secondly anti-inflammatory in rheumatoid arthritis *in vitro* 88. Wygrecka et al. indicate that the ENO1 act as a plasminogen receptor (PLGR), which could promote invasion of monocytes into lungs in mice and humans 19. Further, they find that translocation of ENO1 can be mediated by Ca^2+^ through SOC channels related proteins. Stromal interaction molecule 1 (STIM1)/calcium release-activated calcium channel protein 1 (ORAI1) axis regulate LPS-induced translocation of ENO1 to the cell membrane surface. And, ENO1 is released into the extracellular space in the form of exosomes, and exosomal ENO1 associate with the metastatic potential of cancer cells 89. ENO1 also plays a vital role in urokinase receptor (uPAR)/integrins pathways in pancreatic ductal adenocarcinoma. The knockdown of ENO1 in CFPAC-1 cells results in a markedly increasing expression of uPAR. The binding of uPAR with VN triggers alpha v/beta 3 integrin activates serine/threonine kinase 1-2 (ERK) and Rac family small GTPase to accumulate ROS produce and senescence of pancreatic ductal adenocarcinoma (PDA) cells 90.

As alternatively spliced nuclear isoform of the *ENO1* gene, the expression of MBP-1 is regulated by the AKT/eIF2α axis to response cell stress. The study implies that mRNA alternative splicing of *ENO1* could be controlled by the AKT/eIF2α axis 36. In additional, MBP-1 suppresses tumorigenesis and regulates the development and metastasis of cancer cells by regulating genes expression, including c-Myc, COX-2 and ERBB2 91. Moreover, MBP-1 regulates prostate cancer cell growth by inducing proteasome mediated degradation of the MEK5 to inhibit the MEK5/MAPK7-mediated activation of MEF2C and NF-κB 92. However, the mechanism of alternative splicing changes in the gene of *ENO1* remain unclear in the cell stress response.

## Targeting ENO1 in cancer therapy

The identification of oncogenic ENO1 has triggered the development of small molecule inhibitors and others agents targeting ENO1 for cancer treatment. The group of Florian L Muller have developed a serial of ENO1 inhibitors which perform antitumor activity *in vitro* and *in vivo*.

### ENOblock

**Figure 4:**
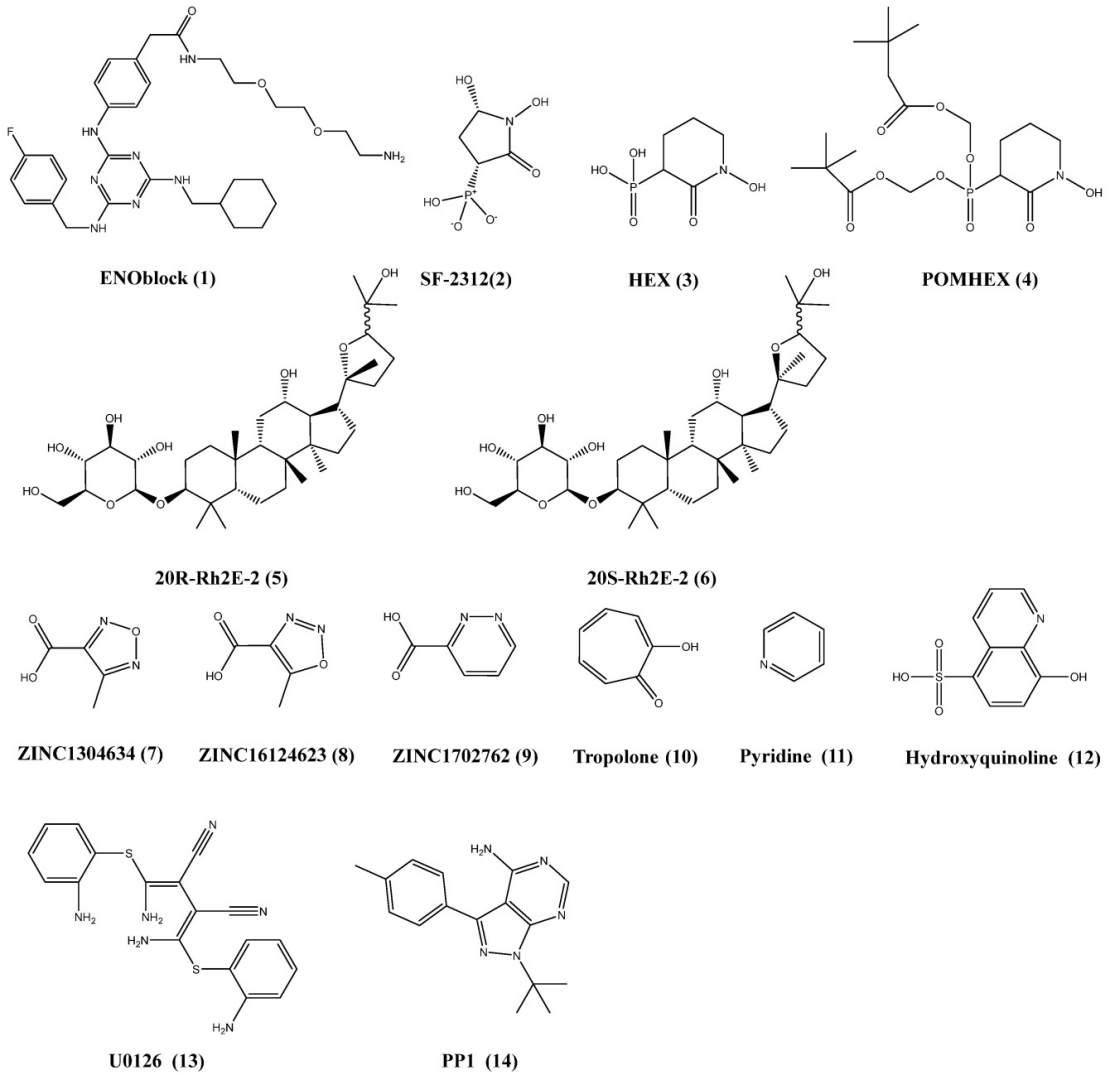
Chemical structures of the chemicals target to the ENO1 and the related pathways.

ENOblock 1 (Figure 4), non-glycolytic inhibitor of ENO1, induce nuclear translocation of ENO1 and down-regulation of phosphoenolpyruvate carboxy kinase to inhibit the growth and migration of cancer cells 7,93,94. Haaglim et al. identify that **1** can alleviate the pathology of diet-induced obesity by regulating the expression of srebp-1a and srebp-1c, PCK1, TNF-α and IL-6 95. In our pervious study, granulin A increased glucose uptake of cells to inhibit the growth of HCC 94,96.

### Derivatives of phosphonoacetohydroxamate

The substrate PEP coordinates with only one Mg^2+^ ion of ENO1 in the active site. Florian L Muller et al. develop a serious of derivatives from natural product SF-2312 2 (Figure 4) which mimics the substrate phosphate group of 2-PGA. And the **2** perform the inhibition of glioma cells growth 23. HEX 3 and POMHEX 4 (Figure 4) is substrate-competitive enolase inhibitor with a Ki of 63 nM for ENO2 versus 250 nM for ENO1 20. Mechanically, **4** diffuses into cell rapidly, and is hydrolyzed by high intracellular carboxylesterase activity and is trapped in the negatively charged mono-ester form. The mono-ester form of **4** is converted to the fully active through the action of phosphodiesterases-4 to inhibit the proliferation of cells. Moreover, the ATP of glioma cells is reduced by **4**, which indicate that **4** can block glycolysis in the tumor. As inhibitor of ENO1, the derivatives of **2** preferentially inhibits glycolysis in ENO1-null over isogenic ENO1-rescued glioma cells. ENO1-intact glioma cells can recover more quickly from enolase inhibition. **4** can eradicate intracranial orthotopic ENO1-deleted GBM tumors in mouse, despite sub-optimal pharmacokinetic properties. In addition, overexpression of ENO1 have been found in many cancer types. ENO1 is more important than other isoform in the glycolysis, and should be a potential target for cancer treatment.

### The ginsenoside derivatives

Huang et al. synthesizes two *ginsenoside* derivatives, 20(R)-Rh2E2 5 and 20(S)-Rh2E2 6, which are structurally modified from parental compound 20(R/S)-Rh2 97,98. The *ginsenoside* derivatives could inhibit the growth of cancer *in vitro* and *in vivo* by energy metabolism intervention, and the tumor bearing mouse are administrated with 20(S)-Rh2E2 up to 320 mg/kg/day and survived with no observable toxicity. Mechanically, the *ginsenoside* derivatives could down-regulated the expression of ENO1 in the cancer cells. And the growth of glycolysis and inhibition of invasion is the result of ENO1 downregulation in the cancer. The results shown that **5** and **6** might be the metabolic inhibitors of cancer with low toxicity for normal cells.

### The other compounds trageting ENO1

The library of compounds has been screening by docking, and some chemicals show better binding energies values than the substrate 2-PG in computer-aided identification of potential tool. These compounds such as ZINC1304634 7, ZINC16124623 8, ZINC1702762 9, tropolone 10, pyridine 11 and hydroxyquinoline 12 (Figure 4) may be considered promising anticancer agents for further development, and could fight the metabolism of cancer by inhibiting ENO1 99,100. Furthermore, upregulation of ENO1 by CagA can be attenuated by U0126 13 (selective inhibitor of both MEK1/2) and PP1 14 (Figure 4) (inhibitor of Src kinase). As shown above, ENO1 act as upstream or downstream regulators in the important signalling. The therapeutic inhibitor targeting to the ENO1 might offer the promising new treatment options for cancer treatment.

## ENO1 in the immunotherapies of cancer

ENO1 and antibody of ENO1 in the serum is associated with the clinical stage of cancer. The expression of ENO1 and antibody of ENO1 in the serum can be detected in some of oral squamous cell carcinoma patients 31,101. Meanwhile, there is a positive correlation between auto-antibody levels of ENO1 and expression of phosphorylated ENO1 or ENO1 in tumor tissue, suggesting that ENO1 might be a biomarker of cancer 16,102-105. The ENO1 are upregulation in the majority of clinically relevant cancers, and serum ENO1 auto-antibody have been reported in wide range of cancers. Zhang et al. demonstrate that serum ENO1 auto-antibody levels is associated with the clinical stage of lung cancer in the patients 104. The other study implies that serum ENO1 antibody levels are significantly higher in the lung cancer than those in the control or in the benign lung disease groups (*P* < 0.001). Serum ENO1 auto-antibody levels in the benign lung disease group is significantly higher than those in the control group (*P* < 0.05). Serum ENO1 auto-antibody levels in the patients in stages I and II were significantly higher than those in the patients with advanced stages III and IV *(P* < 0.01). These result show that auto-antibody level of ENO1 is associated with the clinical stage of lung cancer. But the detail mechanism is remaining unclear. The another study also identify that the decreasing of serum ENO1 antibody is a marker in late stage of NSCLC, SCLC and breast cancers 28. The levels of ENO1 auto-antibody is significantly decreased in both Stage IV of NSCLC and SCLC when compared with those of the normal individuals (*P* < 0.001). In Stage IV breast cancer, the data show that the patients have dramatically lower levels of the ENO1 auto-antibody (*P* < 0.001) than healthy individuals (n = 99) or female controls (n = 49). The study indicates that the titer change of ENO1 is a key factor associating with tumor malignancy of NSCLC, SCLC and breast cancer patients 28. The ENO1 may be a potential marker for the cancer immunotherapy.

The Sun et al. have shown the expression of ENO1 is positively associated with Ki67 expression in the tumor tissues 106. However, the expression of ENO1 is negatively correlated with p53 expression in the tumor tissues. And expression of ENO1 is significantly associated with unfavorable survival. The level of ENO1 (HR=2.469; 95% CI: 1.348-4.522; *P*=0.003) is an independent prognostic for pancreatic patients by Cox multivariate regression analysis. The DNA vaccination of ENO1 could slow progression of PDA by promoting the respond of Th17 T cell, while inhibits expansion of MDSC and Treg cell in the PDA tumor-bearing mice 107. In recently, both DNA vaccination of ENO1 and the combination of DNA vaccination of ENO1 and gemcitabine treatment have shown a significant reduction of PDA lesions compared with untreated in the pancreatic cancer-prone KC mice. They find that the combination of DNA vaccination of ENO1 and gemcitabine shows a significant reduction of circulating CD4 and CD8 T cells, which supports the increasing infiltration of these cells in tumor lesions in the pancreatic cancer-prone KC mice. And CT also could increase the coordinated immune response to DNA vaccination of ENO1 and the combination treatment in the KC mice with PDA 108.

In recently, the preclinical evidence demonstrates that targeting ENO1 induces plasmacytoid dendritic cells triggered T and NK cell mediated anti-multiple myeloma activity 99. Arghya et al. indicate that ENO1 inhibitor could increase plasmacytoid dendritic cells to activate multiple myeloma specific CD8+ T cells and NK cell against autologous tumor cells. As well as the combination of ENO1 inhibitor and anti-PD-L1 antibody or HDAC6 inhibitor synergistically enhances the activity of autologous multiple myeloma specific CD8+ T cells 109. The further research indicats that modification peptides deriving from ENO1 protein can stimulate the CD8+ T cells and CD4+ T cells, peripheral blood mononuclear cells (PBMCs) and tumor infiltrating lymphocytes (TILs) to release cytokine, such as like IFN-γ, TNF-α, as well as the expression of activation marker like CD107a 31. The vaccine targeting to ENO1 have shown encouraging results in the animal model 32.

Cook Katherine et al. indicate targeting post translation modified epitope of ENO1 may provide a new strategy for generation tumor specific immune responses. They show that citrullinated epitopes from ENO1 can be used in vaccines to induce potent CD4^+^ T cell responding to an anti-tumor effect with minimal cross reactivity to healthy tissue. Transgenic mice model demonstrate that immunization with citrullinate ENO1 peptide can simultaneously induce CD4-mediated IFN-γ responses. The citrullinated ENO1 peptides provides a significant advantage survival in the mice model. The study also implies that the CD4^+^ T cells indirect recognize tumor, and direct recognition of the citrullinated epitopes on MHC class II in the tumor is more potent for cancer therapy. Moreover, the studies also identify that healthy human has a repertoire of CD4^+^ T cells capable of responding to the citrullinate ENO1 peptide. Citrullinated ENO1 epitope can be used to generate strong Th1 responses that can preferentially target tumor cells 32. The studies of ENO1-related immune respond in the cancer is a promised thought for cancer immune therapeutics. In the further, the ENO1 related immune in the cancer development should be further investigated to assess the immunotherapies of cancer.

## Conclusion and future perspective

ENO1 is almost 47.2 kilo Dalton. With other two isozyme subunits ENO2 and ENO3, ENO1 could form homodimers heterodimers. The alternative splicing of ENO1 mRNA results in an isoform protein that could binding with the promotor of c-myc to regulating the cell proliferation. The composition of the secondary structure in ENO1 are 21 α-helices, 13 β-sheets, and 4 random coils. Large number of evidences the ENO1 plays a critical role in human cancer and is inappropriately activated in a large fraction of cancers through a variety of different mechanisms. Metabolic reprogramming of tumor associate with anabolic glucose metabolism enzyme, such as ENO1, PGD, GLU1, PKM1/2 and so on. ENO1 can dramatically disparately responds on the cell stress and induces signaling reprogramming due to the distinct change of ENO1, such as alternative splicing, translocation, PTMs of ENO1 and so on. Increased ENO1 expression is detected in major of tumor tissues, and is positive correlation with multi-drug-resistance of cancer cell and poor survive of patients. In recent years, comprehensive biochemical and functional studies have elucidated that ENO1 is a key target for therapeutic development.

Alternative splicing of ENO1 may be an exquisite sense for cancer development. In contrast, translocation of ENO1 and over-expression of ENO1 on the membrane can promote tumor development and metastasis. Inflammatory stimulation also induces translocation of ENO1 from the cytosolic to cell membrane. The switching of ENO1 location is related with the pathology of cancer development and inflammatory 19. The transcription, translation, PTMs of ENO1 and even translocation alteration of ENO1 is associated with the rearrange of cellular process. However, the mechanism of how ENO1 transfer in the cell surface remains unclear, and which factor is the required for cancer development should be further authenticated. So that the future clinical trials of novel strategies targeting ENO1 should include careful pharmacodynamics assessment, through paired pretreatment and on-treatment tumor biopsies to ascertain the specific transduction cascades effects of each therapy on its target. The ENOl server as a regulation factor in the several pathways, such as hypoxia pathway, MAPK/ERK, PI3/AKT, AMPK/mTOR, Wnt/β-catenin pathway and so on. The LncRNA also regulates the transcription and enzyme activity of ENO1 in the cancer. In the tumor environment, the ENO1 may be vital for T cells for competition glucose with cancer cells. Furthermore, the citrullinated ENO1 peptide can be used to generate strong Th1 responses that can preferentially target to tumor cells. Therefore, a detailed understanding of the transcription, translation, translocation, PTMs and unique transduction cascades characteristics of specific ENO1 alterations will be critical to guide the design of effective therapies for cancer treatment.

## Figures and Tables

**Figure 1:**
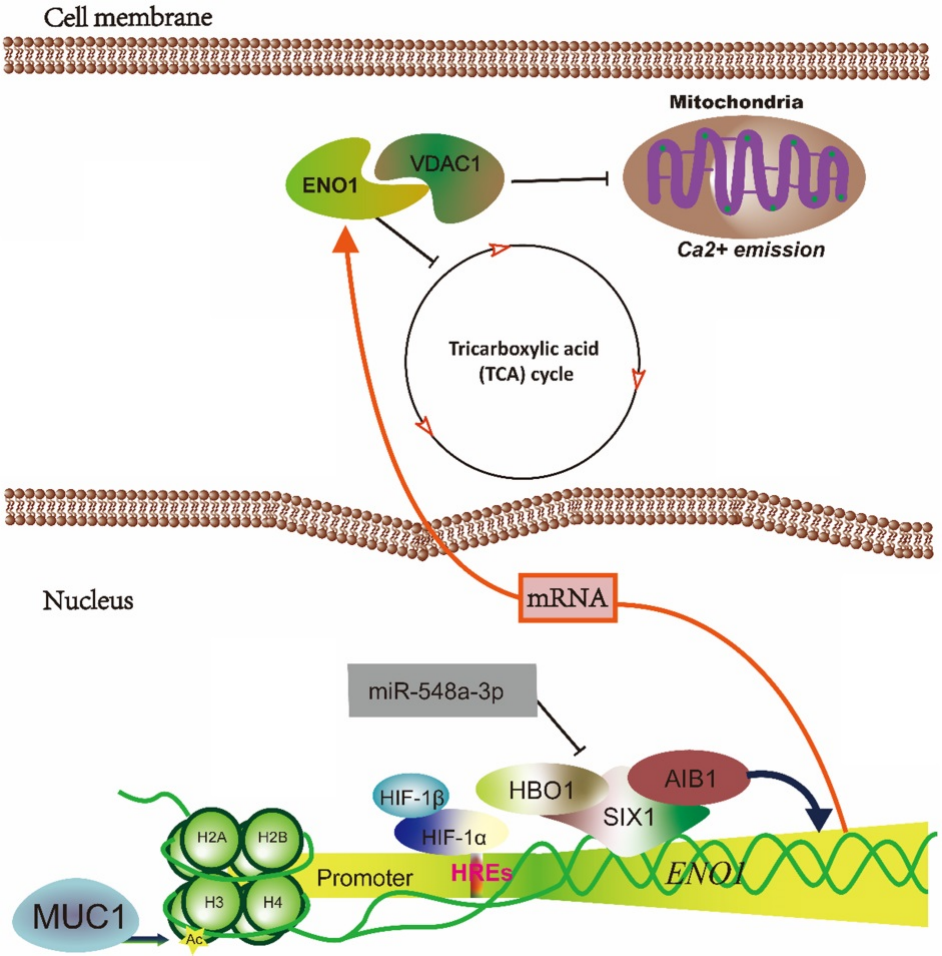
** The schematic diagram illustrated that hypoxia-induced ENO1 expression.** The mRNA of *ENO1* is transcriptionally regulated by MUC1, HIFs and SIX1. And ENO1 could inhibit the TCA cycle and binds with VDAC1 to prevents the Ca^2+^-induced loss of mitochondrial transmembrane potential, swelling of matrix and re-lease of cytochrome c.

**Figure 2:**
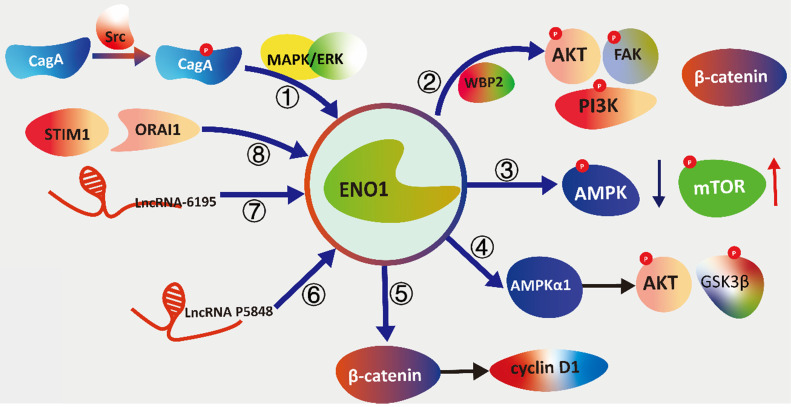
** The schematic diagram illustrating the signal transduction cascades of ENO1 with different pathways.** (①) Cytotoxin-associated gene A (CagA) activates the Src and MAPK/ERK to induce the elevation of expression of ENO1. (②)WW Domain Binding Protein 2 (WBP2) modulating the expression and glycolysis activity of ENO1 to phosphorylated focal adhesion kinase (FAK), PI3K, and AKT. (③) ENO1 inhibits phosphorylation of AMPKα while increases the phosphorylation of mTOR concomitantly to promote tumorigenesis and metastasis. (④) ENO1 is overexpression to regulate proliferation, de-differentiation, resistance to apoptosis and the hypox-ia-induced metabolic shift via the AMPKα1/AKT/GSK3β. (⑤) ENO1 can up-regulate β-catenin as well as its downstream targets cyclin D1 to promotes cell growth and proliferation. (⑥) LncRNA P5848 promotes HCC development by upregulating ENO1. (⑦) LncRNA-6195 reduces enzymatic activity of ENO1 to represses the growth of HCC via directly binding with ENO1. (⑧) Stromal interaction molecule 1 (STIM1)/ calcium release-activated calcium channel protein 1 (ORAI1) axis reg-ulate LPS-induced translocation of ENO1 to the cell membrane surface.

**Figure 3:**
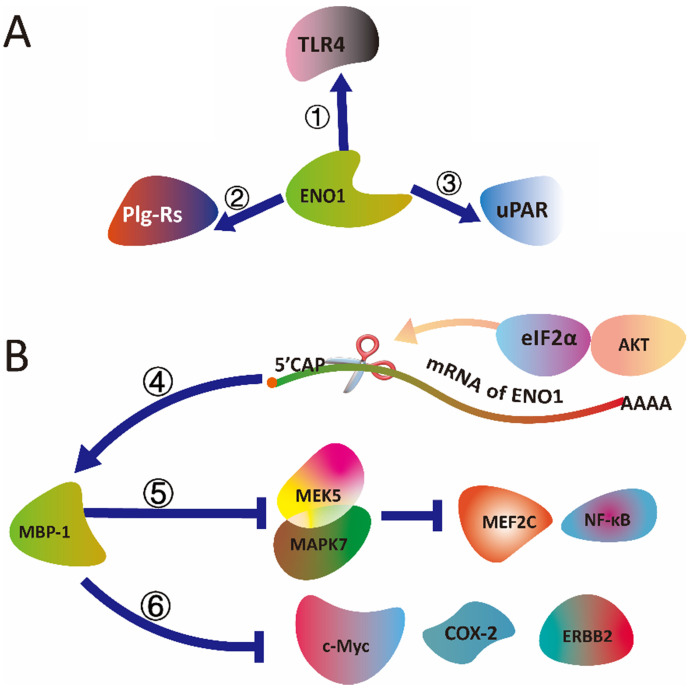
** The schematic diagram illustrated that (A) ENO1 binding with TLR4, uPAR and Plg-Rs, and (B) MBP-1 (ENO1) mediated signal transduction.** (①) ENO1 is able to activate the CD14-dependent TLR4 pathway via binding with TLR4 on monocytes involving in a dual mechanism firstly pro-inflammatory and secondly anti-inflammatory. (②) As a plasminogen receptor (PLGR), ENO1 could binding with Plg-Rs to promote invasion of monocytes into lungs in mice and humans (③) ENO1 interact with uPAR to activates serine/threonine kinase 1-2 (ERK) and Rac family small GTPase to accumulate ROS produce and senescence of pancreatic ductal adenocarcinoma cells. (④) alternative splicing of ENO1 mRNA could be controlled by the AKT/eIF2α axis. (⑤) MBP-1 regulates prostate cancer cell growth by inducing proteasome mediated degradation of the MEK5 to inhibit the MEK5/MAPK7-mediated activation of MEF2C and NF-κB. (⑥) MBP-1 suppresses tumorigenesis and regulates the development and metastasis of cancer cells by regulating genes expression, including c-Myc, COX-2 and ERBB2.

**Table 1:** The post-modification residues position and the function of ENO1

Ontology	Residues position(s)	Reference	Function change
N-acetylserine	2	Jacome et al. (43)	unknown
N6-acetyllysine	5, 60, 64, 71, 89, 92, 126, 193, 199, 202, 228, 233, 256, 257, 281, 283, 285, 326, 335, 343, 406, 420	Choudhary et al. (44); Nakayasu et al. (45); Giannakopoulos et al. (46)	Substrate binding capacity
N6-succinyllysine	60, 80, 81, 89, 228,335, 420	Annapoorna Sreedhar et al. (47); Dong et al. (48); Kiran et al. (49)	Catalyst activity
Phosphoserine	27, 115, 282, 254, 263, 272, 282, 291	Dephoure et al. (50); Zhou et al. (51); Bian et al.(52); Mayya et al. (53); Terytty Yang et al. (54)	Cellular glycolysis
Phosphotyrosine	44, 287	Rush et al. (55). Ross SH et al. (56)	Cytotoxic T cells capacity
N6-malonyllysine	233, 420	Peng et al. (57); Nishida et al. (58).	unknown
Initiator methionine removed	1	Bienvenut et al. (59).	unknown
Arginine methylation	50	Zakrzewicz et al. (30).	Cell invasion and migration
Khib	58, 228, 281, 343	Huang et al. (60), Dong et al. (48).	Cellular glycolysis
Phosphoglycerylation-lysine	343	Moellering et al. (61)	Cellular glycolysis
Citrullination	15, 253	Katherine Cook et al. (32)	Auto-immune
Ubiquitin-like modifiers	Unknow	Giannakopoulos et al. (46); Wong et al. (62); Peng et al. (63)	unknown

## Abbreviations

2-PGA: 2-Phosphoglyceric acid
AIB1: Nuclear receptor coactivator 3
AKT: Protein kinase B
AKT: Protein kinase B
AMP: Adenosine monophosphate
AMPK: AMP-activated protein kinase
ATP: Adenosine triphosphate
CagA: Cytotoxin-associated gene A
CBR2: Carbonyl reductase
c-Myc: MYC Proto-Oncogene
CobB: NAD-dependent protein deacylase
CPT1A: Carnitine palmitoyltransferase I
eIF2α: Eukaryotic Initiation Factor 2
ENO1: Enolase 1
ERK: Serine/threonine protein kinase
ERK1: Serine/threonine kinase 1
FAK: Focal adhesion kinase
GBM: Glioblastoma
GSK3β: Glycogen synthase kinase 3 beta
HBO1: acetyltransferase
HDAC11: Histone deacetylase 11
HDAC6: Histone Deacetylase 6
HIF1: Hypoxia-inducible factor 1
HLA: human leukocyte antigen
HREs: Hypoxia-response elements
IL: Interleukin
Khib: hydroxyisobutyrylation
lncRNA: Long non-coding RNA
LPS: Lipopolysaccharides
MAPK: Mitogen-activated protein kinase
MAPK7: Mitogen-Activated Protein Kinase Kinase 7
MBP-1: c-myc binding protein
MEF2C: Myocyte Enhancer Factor 2C
MEK5: Mitogen-Activated Protein Kinase Kinase 5
MHC: Major histocompatibility complex
mTOR: Mammalian target of rapamycin
MUC1: Mucin-1
NF-κB: Nuclear factor-κB
NSCLC: Non-small-cell lung carcinoma
ORAI1: Calcium release-activated calcium channel protein 1
PASMC: Human Pulmonary Artery Smooth Muscle Cells
PBMCs: Peripheral blood mononuclear cell
PCK1: Phosphoenolpyruvate Carboxykinase 1
PDIA3: Protein Disulfide Isomerase Family A Member 3
PD-L1: Programmed death-ligand 1
PEP: Phosphoenolpyruvic acid
pgK: Phosphoglycerate kinase
PI3K: Phosphoinositide 3-kinase
PLG: plasminogen
PLGR: plasminogen receptor
POMHEX: Esters of (S)-(1-hydroxy-2-oxopiperidin-3-yl)phosphonate
POMSF: (((1-(benzyloxy)-5-hydroxy-2-oxopyrrolidin-3-yl)phosphoryl)bis(oxy))bis (methylene)bis(2,2-dimethylpropanoate)
PRMT5: Protein arginine methyltransferase 5
PTMs: Post-translational modifications
SCLC: Small cell lung cancer
SIRT5: Sirtuin 5
SIX1: Sineoculis homeobox homolog 1
Srebp: Sterol regulatory element-binding proteins
STIM1: Stromal Interaction Molecule 1
T1α: podoplanin
TCA: Tricarboxylic acid
TCR: T cell receptor
TILs: Tumor-infiltrating lymphocytes
TLR4: Toll Like Receptor 4
TNF: Tumor Necrosis Factor
ULK1: Serine/threonine-protein kinase
uPAR: Urokinase receptor
VDAC1: Voltage-dependent anion-selective channel 1
VN: vitronectin
WBP2: WW Domain Binding Protein 2

## References

[1] Kang HJ, Jung SK, Kim SJ, Chung SJ (2008). Structure of human alpha-enolase (hENO1), a multifunctional glycolytic enzyme. Acta Crystallogr, SectD.

[2] Aguayo-Ortiz R, Meza-Cervantez P, Castillo R, Hernández-Campos A, Dominguez L, Yépez-Mulia L (2017). Insights into the Giardia intestinalis enolase and human plasminogen interaction. Mol Biosyst.

[3] Castello A, Fischer B, Eichelbaum K, Horos R, Beckmann BM, Strein C (2012). Insights into RNA biology from an atlas of mammalian mRNA-binding proteins. Cell.

[4] Feo S, Arcuri D, Piddini E, Passantino R, Giallongo A (2000). ENO1 gene product binds to the c-myc promoter and acts as a transcriptional repressor: relationship with Myc promoter-binding protein 1 (MBP-1). FEBS Lett.

[5] Entelis N, Brandina I, Kamenski P, Krasheninnikov IA, Martin RP, Tarassov I (2006). A glycolytic enzyme, enolase, is recruited as a cofactor of tRNA targeting toward mitochondria in Saccharomyces cerevisiae. Genes Dev.

[6] Chaves ML, Camozzato AL, Ferreira ED, Piazenski I, Kochhann R, Dall'Igna O (2010). Serum levels of S100B and NSE proteins in Alzheimer's disease patients. J Neuroinflammation.

[7] Cho H, Um J, Lee J-H, Kim W-H, Kang WS, Kim SH (2017). ENOblock, a unique small molecule inhibitor of the non-glycolytic functions of enolase, alleviates the symptoms of type 2 diabetes. Sci Rep.

[8] Chaparro-Huerta V, Flores-Soto ME, Merin Sigala ME, Barrera de León JC, Lemus-Varela M de L, Torres-Mendoza BM de G (2017). Proinflammatory Cytokines, Enolase and S-100 as Early Biochemical Indicators of Hypoxic-Ischemic Encephalopathy Following Perinatal Asphyxia in Newborns. Pediatr Neonatol.

[9] Mjønes P, Sagatun L, Nordrum IS, Waldum HL (2017). Neuron-Specific Enolase as an Immunohistochemical Marker is better than its Reputation. J Histochem Cytochem.

[10] Schnell SA, Ambesi-Impiombato A, Sanchez-Martin M, Belver L, Xu L, Qin Y (2015). Therapeutic targeting of HES1 transcriptional programs in T-ALL. Blood.

[11] Koshkin VS, Dhawan A, Hu M, Reynolds J, Elson P, McKenney J (2018). Correlation between gene expression and prognostic biomarkers in small cell bladder cancer (SCBC). JCO.

[12] Zhu X, Yu H, Li B, Quan J, Zeng Z, Li G (2018). Targeting a LncRNA P5848-ENO1 axis inhibits tumor growth in hepatocellular carcinoma. Biosci Rep.

[13] Yu S, Li N, Huang Z, Chen R, Yi P, Kang R (2018). A novel lncRNA, TCONS_00006195, represses hepatocellular carcinoma progression by inhibiting enzymatic activity of ENO1. Cell Death Dis.

[14] Huang Z, Lin B, Pan H, Du J, He R, Zhang S (2019). Gene expression profile analysis of ENO1 knockdown in gastric cancer cell line MGC-803. Oncol Lett.

[15] Zhang L, Wang H, Dong X (2018). Diagnostic value of α-enolase expression and serum α-enolase autoantibody levels in lung cancer. J Bras Pneumol.

[16] Chang G-C, Liu K-J, Hsieh C-L, Hu T-S, Charoenfuprasert S, Liu H-K (2006). Identification of alpha-enolase as an autoantigen in lung cancer: its overexpression is associated with clinical outcomes. Clin Cancer Res.

[17] Song Y, Luo Q, Long H, Hu Z, Que T, Zhang X (2014). Alpha-enolase as a potential cancer prognostic marker promotes cell growth, migration, and invasion in glioma. Mol Cancer.

[18] Soni S, Padwad YS (2017). HIF-1 in cancer therapy: two decades long story of a transcription factor. Acta Oncologica.

[19] Wygrecka M, Marsh LM, Morty RE, Henneke I, Guenther A, Lohmeyer J (2009). Enolase-1 promotes plasminogen-mediated recruitment of monocytes to the acutely inflamed lung. Blood.

[20] Lin Y-H, Satani N, Hammoudi N, Pisaneschi F, Leonard P, Maxwell D (2017). Abstract A39: Pomhex, a cell-permeable high potency enolase inhibitor with utility for collateral lethality treatment of cancer. Mol Cancer Ther.

[21] Lin Y-H, Satani N, Hammoudi N, Yan VC, Barekatain Y, Khadka S (2020). An enolase inhibitor for the targeted treatment of ENO1 -deleted cancers. Nat Metab.

[22] Yang T, Shu X, Zhang H-W, Sun L-X, Yu L, Liu J (2020). Enolase 1 regulates stem cell-like properties in gastric cancer cells by stimulating glycolysis. Cell Death Dis.

[23] Leonard PG, Satani N, Maxwell D, Lin Y-H, Hammoudi N, Peng Z (2016). SF2312 is a natural phosphonate inhibitor of Enolase. Nat Chem Biol.

[24] Ye Y, Kuhn C, Kösters M, Arnold GJ, Ishikawa-Ankerhold H, Schulz C (2019). Anti α-enolase antibody is a novel autoimmune biomarker for unexplained recurrent miscarriages. EBioMedicine.

[25] Godier A, Hunt BJ (2013). Plasminogen receptors and their role in the pathogenesis of inflammatory, autoimmune and malignant disease. J Thromb Haemost.

[26] Gemta LF, Siska PJ, Nelson ME, Gao X, Liu X, Locasale JW (2019). Impaired enolase 1 glycolytic activity restrains effector functions of tumor-infiltrating CD8+ T cells. Sci Immunol.

[27] Cappello P, Tomaino B, Chiarle R, Ceruti P, Novarino A, Castagnoli C (2009). An integrated humoral and cellular response is elicited in pancreatic cancer by alpha-enolase, a novel pancreatic ductal adenocarcinoma-associated antigen. Int J Cancer.

[28] Shih N-Y, Lai H-L, Chang G-C, Lin H-C, Wu Y-C, Liu JM (2010). Anti-alpha-enolase Autoantibodies Are Down-regulated in Advanced Cancer Patients. Jpn J Clin Oncol.

[29] Forster F, Paster W, Supper V, Schatzlmaier P, Sunzenauer S, Ostler N (2014). Guanylate Binding Protein 1-Mediated Interaction of T Cell Antigen Receptor Signaling with the Cytoskeleton. J Immun.

[30] Zakrzewicz D, Didiasova M, Krüger M, Giaimo BD, Borggrefe T, Mieth M (2018). Protein arginine methyltransferase 5 mediates enolase-1 cell surface trafficking in human lung adenocarcinoma cells. Biochim Biophys Acta Mol Basis Dis.

[31] Chia J-S, Tuan C-H, Lay F-Y, Ye J-H, Chiu Y-L (2019). Anti-alpha-enolase T cell response in oral squamous cell carcinoma. J Immu.

[32] Cook K, Daniels I, Symonds P, Pitt T, Gijon M, Xue W (2018). Citrullinated α-enolase is an effective target for anti-cancer immunity. Oncoimmunology.

[33] Cappello P, Principe M, Bulfamante S, Novelli F (2017). Alpha-Enolase (ENO1), a potential target in novel immunotherapies. Front Biosci (Landmark Ed).

[34] Semenza GL, Jiang B-H, Leung SW, Passantino R, Concordet J-P, Maire P (1996). Hypoxia Response Elements in the Aldolase A, Enolase 1, and Lactate Dehydrogenase A Gene Promoters Contain Essential Binding Sites for Hypoxia-inducible Factor 1. J Biol Chem.

[35] Giallongo A, Venturella S, Oliva D, Barbieri G, Rubino P, Feo S (1993). Structural features of the human gene for muscle-specific enolase. Eur J Biochem.

[36] Maranto C, Perconti G, Contino F, Rubino P, Feo S, Giallongo A (2015). Cellular stress induces cap-independent alpha-enolase/MBP-1 translation. FEBS Lett.

[37] Kang HJ, Jung S-K, Kim SJ, Chung SJ (2008). Structure of human alpha-enolase (hENO1), a multifunctional glycolytic enzyme. Acta Crystallogr D Biol Crystallogr.

[38] Krucinska J, Falcone E, Erlandsen H, Hazeen A, Lombardo MN, Estrada A (2019). Structural and Functional Studies of Bacterial Enolase, a Potential Target against Gram-Negative Pathogens. Biochemistry.

[39] Terrier B, Degand N, Guilpain P, Servettaz A, Guillevin L, Mouthon L (2007). Alpha-enolase: a target of antibodies in infectious and autoimmune diseases. Autoimmun Rev.

[40] Liu H, Zhang Y, Yang W (2000). How Is the Active Site of Enolase Organized To Catalyze Two Different Reaction Steps?. J Am Chem Soc.

[41] Miller DM, Thomas SD, Islam A, Muench D, Sedoris K (2012). c-Myc and Cancer Metabolism. Clin Cancer Res.

[42] Zhou W, Capello M, Fredolini C, Piemonti L, Liotta LA, Novelli F (2010). Mass Spectrometry Analysis of the Post-Translational Modifications of α-Enolase from Pancreatic Ductal Adenocarcinoma Cells. J Proteome Res.

[43] Jacome ASV, Rabilloud T, Schaeffer-Reiss C, Rompais M, Ayoub D, Lane L (2015). N-terminome analysis of the human mitochondrial proteome. Proteomics.

[44] Choudhary C, Kumar C, Gnad F, Nielsen ML, Rehman M, Walther TC (2009). Lysine Acetylation Targets Protein Complexes and Co-Regulates Major Cellular Functions. Science.

[45] Nakayasu ES, Burnet MC, Walukiewicz HE, Wilkins CS, Shukla AK, Brooks S (2017). Ancient Regulatory Role of Lysine Acetylation in Central Metabolism. mBio.

[46] Giannakopoulos NV, Luo J-K, Papov V, Zou W, Lenschow DJ, Jacobs BS (2005). Proteomic identification of proteins conjugated to ISG15 in mouse and human cells. Biochem Biophys Res Commun.

[47] Sreedhar A, Wiese EK, Hitosugi T (2020). Enzymatic and metabolic regulation of lysine succinylation. Genes Dis.

[48] Dong H, Zhai G, Chen C, Bai X, Tian S, Hu D (2019). Protein lysine de-2-hydroxyisobutyrylation by CobB in prokaryotes. Sci. Adv.

[49] Kurmi K, Hitosugi S, Wiese EK, Boakye-Agyeman F, Gonsalves WI, Lou Z (2018). Carnitine Palmitoyltransferase 1A Has a Lysine Succinyltransferase Activity. Cell Rep.

[50] Dephoure N, Zhou C, Villén J, Beausoleil SA, Bakalarski CE, Elledge SJ (2008). A quantitative atlas of mitotic phosphorylation. Proc Natl Acad Sci USA.

[51] Zhou H, Di Palma S, Preisinger C, Peng M, Polat AN, Heck AJR (2013). Toward a Comprehensive Characterization of a Human Cancer Cell Phosphoproteome. J Proteome Res.

[52] Bian Y, Song C, Cheng K, Dong M, Wang F, Huang J (2014). An enzyme assisted RP-RPLC approach for in-depth analysis of human liver phosphoproteome. J Proteomics.

[53] Mayya V, Lundgren DH, Hwang S-I, Rezaul K, Wu L, Eng JK (2009). Quantitative phosphoproteomic analysis of T cell receptor signaling reveals system-wide modulation of protein-protein interactions. Sci Signal.

[54] Li TY, Sun Y, Liang Y, Liu Q, Shi Y, Zhang C-S (2016). ULK1/2 Constitute a Bifurcate Node Controlling Glucose Metabolic Fluxes in Addition to Autophagy. Mol. Cell.

[55] Rush J, Moritz A, Lee KA, Guo A, Goss VL, Spek EJ (2005). Immunoaffinity profiling of tyrosine phosphorylation in cancer cells. Nat Biotechnol.

[56] Ross SH, Rollings C, Anderson KE, Hawkins PT, Stephens LR, Cantrell DA (2016). Phosphoproteomic Analyses of Interleukin 2 Signaling Reveal Integrated JAK Kinase-Dependent and -Independent Networks in CD8+ T Cells. Immunity.

[57] Peng C, Lu Z, Xie Z, Cheng Z, Chen Y, Tan M (2011). The first identification of lysine malonylation substrates and its regulatory enzyme. Mol. Cell Proteomics.

[58] Nishida Y, Rardin MJ, Carrico C, He W, Sahu AK, Gut P (2015). SIRT5 Regulates both Cytosolic and Mitochondrial Protein Malonylation with Glycolysis as a Major Target. Mol. Cell.

[59] UniProt (2017). the universal protein knowledgebase. Nucleic Acids Res.

[60] Huang H, Tang S, Ji M, Tang Z, Shimada M, Liu X (2018). p300-Mediated Lysine 2-Hydroxyisobutyrylation Regulates Glycolysis. Mol. Cell.

[61] Moellering RE, Cravatt BF (2013). Functional lysine modification by an intrinsically reactive primary glycolytic metabolite. Science.

[62] Wong JJY, Pung YF, Sze NS-K, Chin K-C (2006). HERC5 is an IFN-induced HECT-type E3 protein ligase that mediates type I IFN-induced ISGylation of protein targets. Proc Natl Acad Sci USA.

[63] Peng Q-S, Li G-P, Sun W-C, Yang J-B, Quan G-H, Liu N (2016). Analysis of ISG15-Modified Proteins from A549 Cells in Response to Influenza Virus Infection by Liquid Chromatography-Tandem Mass Spectrometry. Chinese J. Anal. Chem.

[64] Sharma V, Christodoulidou A, Yue L, Alontaga AY, Goodheart WE, Hesterberg R (2018). Abstract LB-249: HDAC11 regulates lysine acetylation of enolase 1. Cancer Res.

[65] Narita T, Weinert BT, Choudhary C (2019). Functions and mechanisms of non-histone protein acetylation. Nat Rev Mol. Cell Biol.

[66] Zhu D, Hou L, Hu B, Zhao H, Sun J, Wang J (2016). Crosstalk among proteome, acetylome and succinylome in colon cancer HCT116 cell treated with sodium dichloroacetate. Sci. Rep.

[67] Du J, Zhou Y, Su X, Yu JJ, Khan S, Jiang H (2011). Sirt5 Is a NAD-Dependent Protein Lysine Demalonylase and Desuccinylase. Science.

[68] Zhu W, Jiang X, Sun H, Li Y, Shi W, Zheng M (2020). Global lysine acetylation and 2-hydroxyisobutyrylation reveal the metabolism conversion mechanism in Giardia lamblia. Mol. Cell Proteomics.

[69] Wang Y-P, Lei Q-Y (2018). Metabolite sensing and signaling in cell metabolism. Sig Transduct Target Ther.

[70] Mulvihill MM, Nomura DK (2014). Metabolomic strategies to map functions of metabolic pathways. Am. J. Physiol. Endocrinol. Metab.

[71] Yin J, Cao H, Wang H, Sun K, Li Y, Zhang Z (2020). Phospholipid membrane-decorated deep-penetrated nanocatalase relieve tumor hypoxia to enhance chemo-photodynamic therapy. Acta Pharm Sin B.

[72] Chaika NV, Gebregiworgis T, Lewallen ME, Purohit V, Radhakrishnan P, Liu X (2012). MUC1 mucin stabilizes and activates hypoxia-inducible factor 1 alpha to regulate metabolism in pancreatic cancer. Proc. Natl. Acad. Sci. U.S.A.

[73] Sedoris KC, Thomas SD, Miller DM (2010). Hypoxia induces differential translation of enolase/MBP-1. BMC Cancer.

[74] Masterson JC, Biette KA, Hammer JA, Nguyen N, Capocelli KE, Saeedi BJ (2019). Epithelial HIF-1α/claudin-1 axis regulates barrier dysfunction in eosinophilic esophagitis. J Clin Invest.

[75] Li L, Liang Y, Kang L, Liu Y, Gao S, Chen S (2018). Transcriptional Regulation of the Warburg Effect in Cancer by SIX1. Cancer Cell.

[76] Capello M, Ferri-Borgogno S, Riganti C, Chattaragada MS, Principe M, Roux C (2015). Targeting the Warburg effect in cancer cells through ENO1 knockdown rescues oxidative phosphorylation and induces growth arrest. Oncotarget.

[77] Zhang Y, Sampathkumar A, Kerber SM-L, Swart C, Hille C, Seerangan K (2020). A moonlighting role for enzymes of glycolysis in the co-localization of mitochondria and chloroplasts. Nat. Commun.

[78] Gao S, Li H, Cai Y, Ye J, Liu Z, Lu J (2014). Mitochondrial binding of α-enolase stabilizes mitochondrial membrane: its role in doxorubicin-induced cardiomyocyte apoptosis. Arch. Biochem. Biophys.

[79] Chen S, Duan G, Zhang R, Fan Q (2014). Helicobacter pylori cytotoxin-associated gene A protein upregulates α-enolase expression via Src/MEK/ERK pathway: Implication for progression of gastric cancer. Int. J. Oncol.

[80] Sun L, Lu T, Tian K, Zhou D, Yuan J, Wang X (2019). Alpha-enolase promotes gastric cancer cell proliferation and metastasis via regulating AKT signaling pathway. Eur J Pharmacol.

[81] Chen S, Zhang Y, Wang H, Zeng Y-Y, Li Z, Li M-L (2018). WW domain-binding protein 2 acts as an oncogene by modulating the activity of the glycolytic enzyme ENO1 in glioma. Cell Death Dis.

[82] Fu Q-F, Liu Y, Fan Y, Hua S-N, Qu H-Y, Dong S-W (2015). Alpha-enolase promotes cell glycolysis, growth, migration, and invasion in non-small cell lung cancer through FAK-mediated PI3K/AKT pathway. J. Hematol. Oncol.

[83] Zhan P, Zhao S, Yan H, Yin C, Xiao Y, Wang Y (2017). α-enolase promotes tumorigenesis and metastasis via regulating AMPK/mTOR pathway in colorectal cancer. Mol. Carcinog.

[84] Dai J, Zhou Q, Chen J, Rexius-Hall ML, Rehman J, Zhou G (2018). Alpha-enolase regulates the malignant phenotype of pulmonary artery smooth muscle cells via the AMPK-Akt pathway. Nat. Commun.

[85] Muller FL, Colla S, Aquilanti E, Manzo V, Genovese G, Lee J (2012). Passenger Deletions Generate Therapeutic Vulnerabilities in Cancer. Nature.

[86] Ji M, Wang Z, Chen J, Gu L, Chen M, Ding Y (2019). Up-regulated ENO1 promotes the bladder cancer cell growth and proliferation via regulating β-catenin. Biosci Rep.

[87] Mutze K, Vierkotten S, Milosevic J, Eickelberg O, Koenigshoff M (2015). Enolase 1 (ENO1) and protein disulfide-isomerase associated 3 (PDIA3) regulate Wnt/beta-catenin-driven trans-differentiation of murine alveolar epithelial cells. Dis Model Mech.

[88] Guillou C, Fréret M, Fondard E, Derambure C, Avenel G, Golinski M-L (2016). Soluble alpha-enolase activates monocytes by CD14-dependent TLR4 signalling pathway and exhibits a dual function. Sci. Rep.

[89] Didiasova M, Zakrzewicz D, Magdolen V, Nagaraj C, Bálint Z, Rohde M (2015). STIM1/ORAI1-mediated Ca2+ Influx Regulates Enolase-1 Exteriorization. J Biol Chem.

[90] Principe M, Borgoni S, Cascione M, Chattaragada MS, Ferri-Borgogno S, Capello M (2017). Alpha-enolase (ENO1) controls alpha v/beta 3 integrin expression and regulates pancreatic cancer adhesion, invasion, and metastasis. J. Hematol. Oncol.

[91] Contino F, Mazzarella C, Ferro A, Lo Presti M, Roz E, Lupo C (2013). Negative transcriptional control of ERBB2 gene by MBP-1 and HDAC1: diagnostic implications in breast cancer. BMC Cancer.

[92] Ghosh AK, Steele R, Ray RB (2005). c-myc Promoter-binding protein 1 (MBP-1) regulates prostate cancer cell growth by inhibiting MAPK pathway. J Biol Chem.

[93] Jung D-W, Kim W-H, Park S-H, Lee J, Kim J, Su D (2013). A unique small molecule inhibitor of enolase clarifies its role in fundamental biological processes. ACS Chem Biol.

[94] Chen X, Xu H, Wu N, Liu X, Qiao G, Su S (2017). Interaction between granulin A and enolase 1 attenuates the migration and invasion of human hepatoma cells. Oncotarget.

[95] Cho H, Lee J-H, Um J, Kim S, Kim Y, Kim W-H (2019). ENOblock inhibits the pathology of diet-induced obesity. Sci Rep.

[96] Qiao G, Xu H, Li C, Li X, Farooqi AA, Zhao Y (2018). Granulin A Synergizes with Cisplatin to Inhibit the Growth of Human Hepatocellular Carcinoma. Int J Mol Sci.

[97] Wong VKW, Dong H, Liang X, Bai L-P, Jiang Z-H, Guo Y (2016). Rh2E2, a novel metabolic suppressor, specifically inhibits energy-based metabolism of tumor cells. Oncotarget.

[98] Huang Q, Zhang H, Bai LP, Law BYK, Xiong H, Zhou X (2020). Novel ginsenoside derivative 20( S )-Rh2E2 suppresses tumor growth and metastasis *in vivo* and *in vitro* via intervention of cancer cell energy metabolism. Cell Death Dis.

[99] Ray A, Song Y, Chauhan D, Anderson KC (2019). Preclinical Validation of Alpha-Enolase (ENO1) As a Novel Immunometabolic Target in Multiple Myeloma. Blood.

[100] Lung J, Chen K-L, Hung C-H, Chen C-C, Hung M-S, Lin Y-C (2017). In silico-based identification of human α-enolase inhibitors to block cancer cell growth metabolically. Drug Des Devel Ther.

[101] Ito S, Honma T, Ishida K, Wada N, Sasaoka S, Hosoda M (2007). Differential expression of the human α-enolase gene in oral epithelium and squamous cell carcinoma. Cancer Sci.

[102] Tomaino B, Cappello P, Capello M, Fredolini C, Ponzetto A, Novarino A (2007). Autoantibody Signature in Human Ductal Pancreatic Adenocarcinoma. J Proteome Res.

[103] Tomaino B, Cappello P, Capello M, Fredolini C, Sperduti I, Migliorini P (2011). Circulating Autoantibodies to Phosphorylated α-Enolase are a Hallmark of Pancreatic Cancer. J Proteome Res.

[104] Zhang L, Wang H, Dong X, Zhang L, Wang H, Dong X (2018). Diagnostic value of α-enolase expression and serum α-enolase autoantibody levels in lung cancer. J Bras Pneumol.

[105] Hu T, Liu H, Liang Z, Wang F, Zhou C, Zheng X (2020). Tumor-intrinsic CD47 signal regulates glycolysis and promotes colorectal cancer cell growth and metastasis. Theranostics.

[106] Sun L, Guo C, Cao J, Burnett J, Yang Z, Ran Y (2017). Over-Expression of Alpha-Enolase as a Prognostic Biomarker in Patients with Pancreatic Cancer. Int J Med Sci.

[107] Cappello P, Rolla S, Chiarle R, Principe M, Cavallo F, Perconti G (2013). Vaccination with ENO1 DNA prolongs survival of genetically engineered mice with pancreatic cancer. Gastroenterology.

[108] Mandili G, Curcio C, Bulfamante S, Follia L, Ferrero G, Mazza E (2020). In pancreatic cancer, chemotherapy increases antitumor responses to tumor-associated antigens and potentiates DNA vaccination. J Immunother Cancer.

[109] Ray A, Song Y, Du T, Chauhan D, Anderson KC (2020). Preclinical validation of Alpha-Enolase (ENO1) as a novel immunometabolic target in multiple myeloma. Oncogene.

